# Exposure to air pollution concentrations of various intensities in early life and allergic sensitisation later in childhood

**DOI:** 10.1186/s12890-023-02815-8

**Published:** 2023-12-21

**Authors:** Myriam Ziou, Caroline X. Gao, Amanda J. Wheeler, Graeme R. Zosky, Nicola Stephens, Luke D. Knibbs, Grant J. Williamson, Marita F. Dalton, Shyamali C. Dharmage, Fay H. Johnston

**Affiliations:** 1 Menzies Institute for Medical Research, University of Tasmania, 17 Liverpool Street, Hobart, Tasmania 7000 Australia; 2 School of Public Health and Preventive Medicine, Monash University, Melbourne, Victoria 3004 Australia; 3 Centre for Youth Mental Health, The University of Melbourne, Parkville, Victoria 3052 Australia; 4 Commonwealth Scientific and Industrial Research Organisation (CSIRO) Environment, Aspendale, Victoria 3195 Australia; 5 Tasmanian School of Medicine, University of Tasmania, Hobart, Tasmania 7000 Australia; 6 School of Public Health, The University of Sydney, Camperdown, NSW 2006 Australia; 7 Public Health Research Analytics and Methods for Evidence, Public Health Unit, Sydney Local Health District, Camperdown, NSW 2050 Australia; 8 School of Natural Sciences, University of Tasmania, Sandy Bay, Tasmania 7005 Australia; 9 Allergy and Lung Health Unit, School of Population and Global Health, The University of Melbourne, Carlton, Victoria 3052 Australia

**Keywords:** Allergic sensitisation, Immunoglobulin E, Landscape fires, Child health, Particulate air pollution, Early life, Long-term effects

## Abstract

**Background:**

Evidence on the relationship between air pollution and allergic sensitisation in childhood is inconsistent, and this relationship has not been investigated in the context of smoke events that are predicted to increase with climate change. Thus, we aimed to evaluate associations between exposure in two early life periods to severe levels of particulate matter with an aerodynamic diameter < 2.5 μm (PM_2.5_) from a mine fire, background PM_2.5_, and allergic sensitisation later in childhood.

**Methods:**

We measured specific immunoglobulin E (IgE) levels for seven common aeroallergens as well as total IgE levels in a cohort of children who had been exposed to the Hazelwood coal mine fire, either in utero or during their first two years of life, in a regional area of Australia where ambient levels of PM_2.5_ are generally low. We estimated personal exposure to fire-specific emissions of PM_2.5_ based on a high-resolution meteorological and pollutant dispersion model and detailed reported movements of pregnant mothers and young children during the fire. We also estimated the usual background exposure to PM_2.5_ at the residential address at birth using a national satellite-based land-use regression model. Associations between both sources of PM_2.5_ and sensitisation to dust, cat, fungi, and grass seven years after the fire were estimated with logistic regression, while associations with total IgE levels were estimated with linear regression.

**Results:**

No association was found between the levels of exposure at either developmental stage to fire-related PM_2.5_ and allergic sensitisation seven years after the event. However, levels of background exposure were positively associated with sensitisation to dust (OR = 1.90, 95%CI = 1.12,3.21 per 1 μg/m^3^).

**Conclusions:**

Chronic but low exposure to PM_2.5_ in early life could be more strongly associated with allergic sensitisation in childhood than time-limited high exposure levels, such as the ones experienced during landscape fires.

**Supplementary Information:**

The online version contains supplementary material available at 10.1186/s12890-023-02815-8.

## Introduction

Allergies have been increasing in prevalence globally over the last few decades [1]. Allergic conditions have been shown to directly impact quality of life, including the psychological, educational, professional and social domains of those affected [2, 3]. Allergies are known to be caused by a combination of genetic, lifestyle and environmental factors, although the relative importance of these, and their interactions, is not fully understood [4]. There is robust evidence showing that exposure to air pollution is associated with the development and exacerbation of allergic conditions such as asthma [5, 6] and allergic rhinitis [7]. However, the relationship with allergic sensitisation is inconsistent and relatively scarce [8].

Children have been consistently identified as a subgroup vulnerable to the effects of air pollution for both physiological and behavioural reasons [9]. A systematic review and meta-analysis of birth cohorts found that early childhood exposure to traffic-related air pollutants was related to increased sensitisation to aeroallergens and food allergens [10]. More recently, one study conducted in Puerto Rican school-aged children found that long-term exposure to SO_2_, a component of air pollution linked to fossil fuel combustion, and living in proximity to a major highway were associated with sensitisation to common allergens [11]. However, several other recent studies have found no association, especially with aeroallergens [12–14]. Such knowledge is important to understand the implications of increasing air pollution levels globally and to develop specific intervention strategies. The field is further limited by the fact that chronic exposures to ambient air pollutants in urban areas have been the focus of research, with no study investigating how air pollution from episodic major pollution events, such as landscape fires, relates to allergic sensitisation in children.

**Fig. 1:**
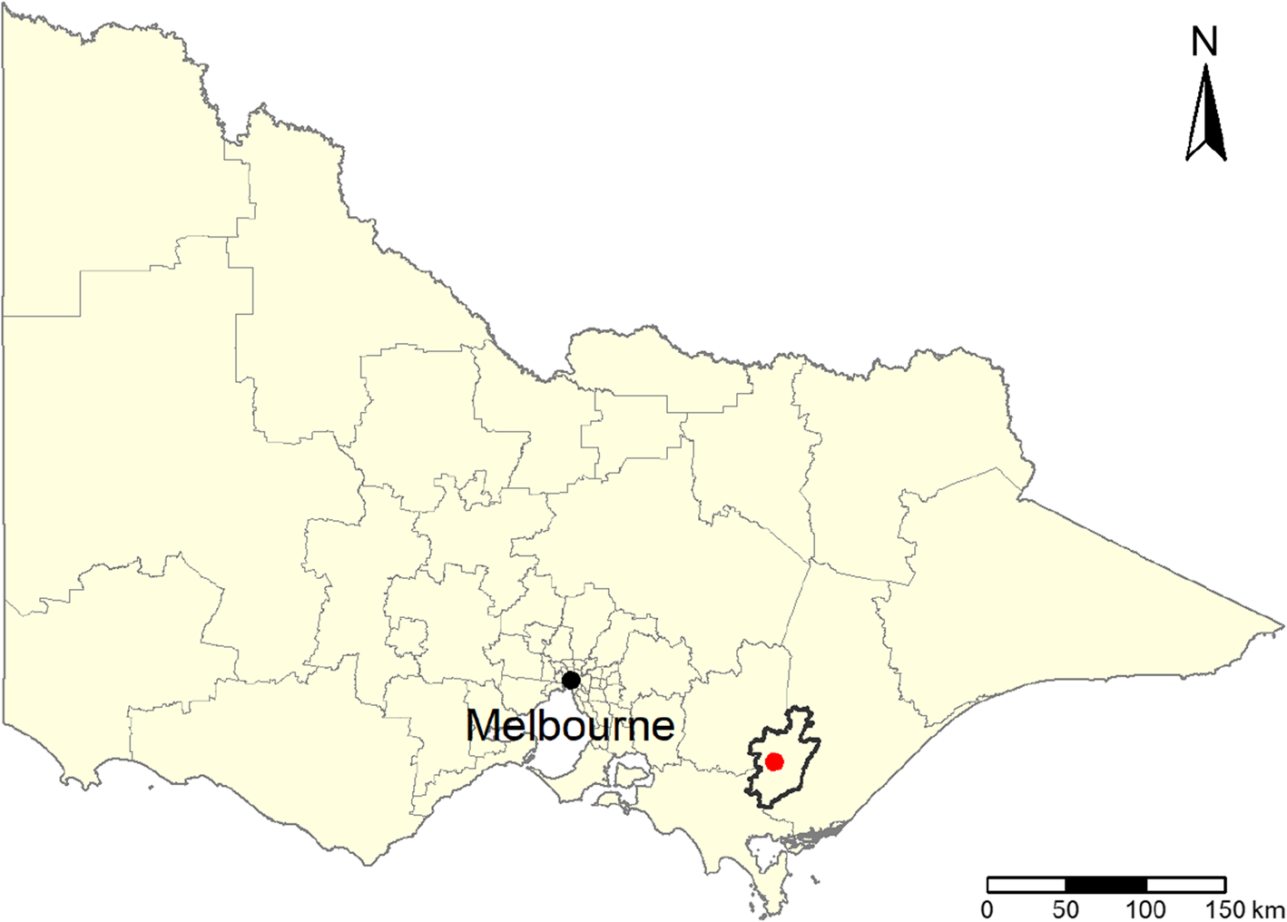
Location of the Latrobe Valley and the Hazelwood mine. Latrobe Valley borders are in bold and the mine location is illustrated as a red dot

In the summer of 2014, a spot fire started in the Hazelwood open-cut brown coal mine in the Latrobe Valley, Victoria, Australia (Fig. 1) leading to an underground fire that burnt for 45 days. Morwell, a town located in the immediate vicinity of the mine, and other localities within the Latrobe Valley, experienced extreme levels of air pollution for several weeks, including elevated particulate matter, carbon monoxide and benzene [15]. The Hazelwood Health Study (HHS) [16, 17] was established to monitor the short- and long-term health and social repercussions in people impacted by the event. As pregnant mothers and young children were identified as a vulnerable subpopulation, the Latrobe Early Life Follow-Up (ELF) Study cohort was established as a stream of the HHS, to evaluate possible long term health and developmental outcomes in children who were either in utero or in their first two years of life at the time of the fire [16]. Previous studies conducted in that cohort found evidence of associations between exposure to PM_2.5_ emissions from the fire and allergic symptoms. Notably, exposure during in utero to was associated with increased wheezing [18] and dispensing of systemic steroids [19], while exposure during infancy was linked to increased use of asthma inhalers [18] and dispensing of steroid skin creams [19]. However, potential pathways behind these observations were unclear.

In this analysis, we leverage that same cohort to address gaps in knowledge regarding the link between short-term, high-intensity air pollution in early life and the subsequent development of allergic sensitisation. Specifically, we aimed to determine whether exposure in prenatal and postnatal periods to air pollution from background sources and the severe smoke event, including long-term exposure to ambient PM_2.5_ (particulate matter with an aerodynamic diameter < 2.5 μm), was associated with subsequent allergic sensitisation determined by serum total Immunoglobulin E (IgE) and allergen-specific IgE production.

## Methods

### Study design

**Fig. 2:**
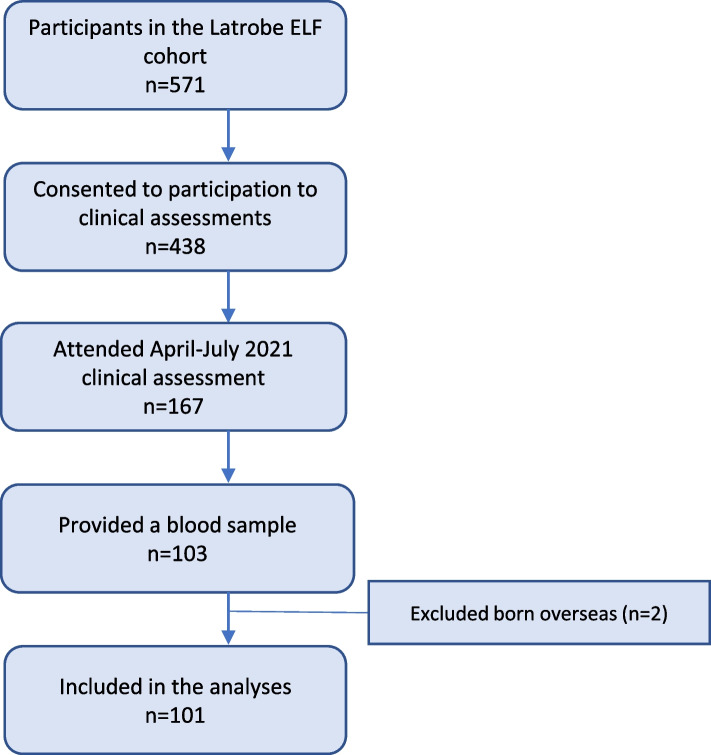
Flowchart of the study participants. ELF: Early Life Follow-Up

The ELF cohort was recruited in 2016 and consisted of 571 children born between 1st March 2012 and 31st December 2015 and residing in the Latrobe Valley at the time of the mine fire. Details regarding the recruitment and characteristics of the ambidirectional cohort have been published elsewhere [16]. Of the 571 children, 438 had a parent or caregiver agreeing at the time of enrolment to participate in longitudinal clinical assessments (three, seven and nine years following the fire) to evaluate respiratory and vascular function. At the second follow-up clinic, held between April and July 2021, the 167 attending participants were also invited to provide a blood sample for markers of allergic sensitisation. Blood samples were collected for 103 children (Fig. 2), with the consent of the participant and the parent or caregiver who provided signed informed consent. This study was approved by the Tasmanian Health and Medical Human Research Ethics Committee (reference H14875).

### Allergic sensitisation

Blood samples were analysed for IgE to seven aeroallergens using chemiluminescent immunoassay technology (Immulite® 2000 XPi, Siemens) by a commercial laboratory (Australian Clinical Labs, Victoria, Australia). The instrument utilised 3gAllergy™ specific allergens and mixed allergen panels (Siemens) [20]. Results were provided in kU/L and participants were classified as sensitised to an allergen if levels of specific IgE to that allergen were ≥ 0.35 kU_A_/L. [21, 22] Aeroallergens assayed included *Alternaria tenuis* (Catalogue No. M6), cat epithelium (Catalogue No. E1), *Cladosporium herbarum* (Catalogue No. M2), *Dermatophagoides pteronyssinus* (house dust mites; HDM; Catalogue No. D1), a dust panel (mix of *D. pteronyssinus*, *D. farinae*, house dust, and cockroach; Catalogue No. HP1), perennial rye grass *(Lolium perenne)* pollen (Catalogue No. G5) and a grass pollen panel (mix of Bermuda, perennial rye, Timothy, Kentucky blue, Johnson, and Bahia grasses; Catalogue No. GP2). For analysis purposes, *A. tenuis* and *Cl. herbarum* were combined under a fungi category, *D. pteronyssinus* and the dust panel under a dust category, and perennial rye grass pollen and the grass pollen panel under a grass category. Combination of allergens within these categories was motivated by (1) the presence of HDM in the dust panel and of perennial rye grass in the grass panel, due to availability of the tests, and (2) the very small number of children sensitised to *Cl. herbarum* within the cohort. Total IgE was measured using a Human IgE ELISA Kit (Catalogue No. BMS2097, Invitrogen) according to manufacturer’s instructions and results were reported as ng/ml. The number of analyses successfully completed for each child (4–8) was dependent on the number of aliquots collected, which varied with their degree of cooperation.

### Exposure assessment

For the purpose of this study, the Hazelwood open-cut coal mine fire was defined as lasting from the 9th February to 28th March 2014, as some low levels of residual smoke remained over certain areas after the fire was declared safe on 26th March 2014. Hourly concentrations of PM_2.5_ emitted specifically by the fire were estimated at a 1-km^2^ resolution by a meteorological and dispersion model incorporating wind data and a plume rise process [23]. Detailed diary reporting 12-hourly locations of the pregnant mother or infant were retrospectively obtained throughout the fire and were used to assign daily average and peak 24-hour average exposures for each child. Prenatal and postnatal exposures were estimated separately for each child depending on their estimated dates of conception and delivery. Only days following the estimated date of conception were considered for children conceived during the fire while those conceived following the fire were assigned average and peak fire-related PM_2.5_ concentrations of 0 μg/m^3^.

To estimate ambient (‘background’) PM_2.5_ exposure, validated satellite-informed land-use regression models were used. The models were constructed with different spatial predictors (e.g.*,* the proportion of households using wood heaters, commercial areas, wind speed), and explained 63% of spatial variation in measured annual PM_2.5_ (RMSE: 1.0 μg/m^3^) across Australia. Details on methodology and validation of the models have been reported elsewhere [24]. Annual averages for the years 2011–2015 of both pollutants were estimated for ∼347,000 census mesh blocks, the smallest geographic areas defined by the Australian Bureau of Statistics [25], throughout the country. Early life background PM_2.5_ exposure was assigned at the mesh block of the birth address by averaging exposure of the years of conception and birth of each child to account for prenatal and early postnatal exposure. There was little year-to-year variation, either within the state of Victoria or within the cohort (Pearson’s *r* > 0.95 for all pairwise correlations between years).

**Fig. 3:**
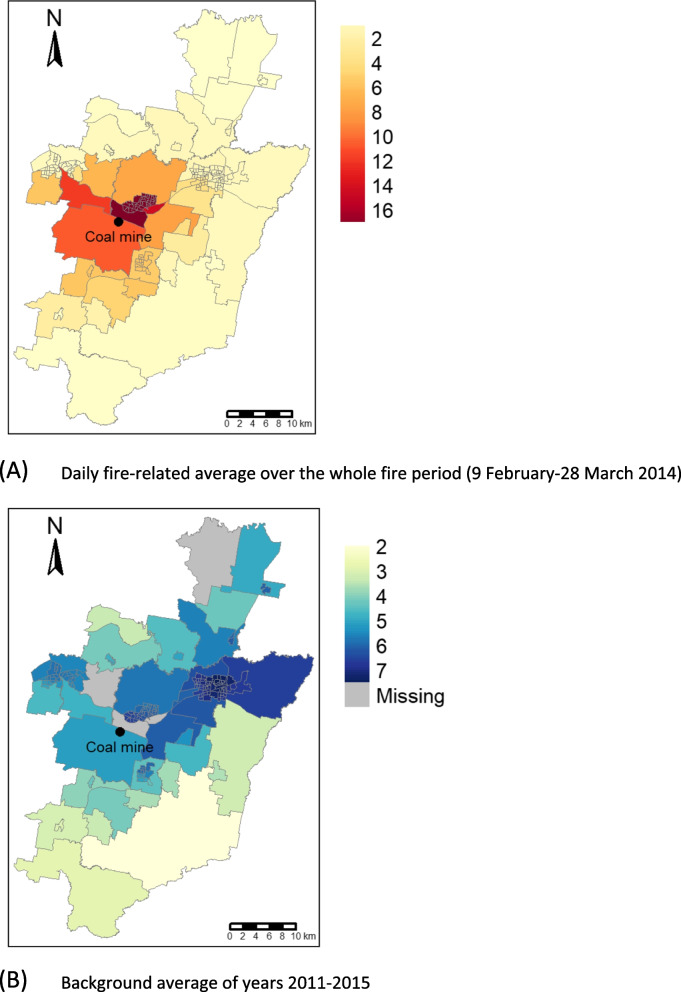
PM_2.5_ concentrations (μg/m^3^) from both sources mapped to Statistical Area level 1 (SA1) [26]

Spatial distribution was different for fire-related and background ambient PM_2.5_ (Fig. 3), which was motivation to investigate them as co-exposures.

### Covariates

During the recruitment in 2016, enrolled participants completed an extensive baseline questionnaire, that included a wide range of characteristics about the child, parents and housing environment [16]. Potential confounding variables were proposed based on the existing literature on air pollution and/or allergic sensitisation or diseases [27–34]. Two minimal sufficient adjustment sets of confounders were identified based on a directed acyclic graph (DAG) using DAGitty v3.0 (Fig. S1) [35] with the intention to estimate the total effect of the exposures on the outcomes. The first one, selected for our primary analysis, included three variables: maternal education (≤ year 12 vs. > year 12), child age in months at the time of the blood collection, and Index of Relative Socio-economic Disadvantage (IRSD) decile of the household. The second included seven variables: breastfeeding (< 6 months vs. ≥ 6 months), main heating type in the house (combustion emissions released vs. not released into the living space), maternal education (≤ year 12 vs. > year 12), pregnancy stress (no/hardly vs. sometimes/mostly), presence of a smoker in the house (yes vs. no), parent with history of asthma or allergic rhinitis, and premature birth. IRSD is an area-level indicator of social and economic disadvantage developed by the Australian Bureau of Statistics incorporating 16 familial, educational, occupational, and financial measurements [36]. It was assigned at the SA1 level, the smallest geographical area defined by the Australian Bureau of Statistics for which census data are released [26], of the home address.

Participants were also asked in the baseline questionnaire “Have you been told by a doctor that the study child has asthma?” and “Have you been told by a doctor that the study child has any eczema or dermatitis?”. However, we chose to not include a past diagnosis of asthma or eczema/dermatitis as a confounder as these two conditions could be on the same causal pathway as allergic sensitisation, and we wanted to avoid adjusting on a potential mediator or collider.

### Statistical analysis

We fitted single- and multi-pollutant logistic regression models to evaluate the exposure-response relationship between the three exposures (prenatal fire-related PM_2.5_, postnatal fire-related PM_2.5_, background PM_2.5_) and (1) sensitisation to each of the allergen categories (fungi, dust, cat, grass), and (2) sensitisation to at least one allergen category. We additionally fitted single- and multi-pollutant linear regression models to estimate the relationship between the air pollutant exposures and total IgE levels. Although total IgE levels are commonly log-transformed in regression analyses [11, 37], the distribution of total IgE was not log-normal within our sample and a log-transformation did not reduce skewness (Fig. S2). Thus, we included non-transformed IgE concentrations in our models. Odds ratios (OR) and β coefficients were estimated per interquartile range (IQR) increase in each pollutant.

Models assessing average and peak fire-related PM_2.5_ exposures were fitted separately. As estimates for background PM_2.5_ were similar in the multi-pollutant models including average and peak metrics, we only presented the estimates with average fire-related PM_2.5_ as a co-exposure. All models fitted were adjusted for the first minimal sufficient adjustment set of confounders.

Multiple imputation by chained equations was performed to handle missing data using the *mice* package (v 3.15.0) [38]. A total of twenty imputed datasets, including all exposure, confounding and outcome variables, was created with a random forest algorithm for continuous and categorical variables. Confounding or outcome variables derived from collected or measured data were imputed following the *Impute, then transform* approach, where primary data is imputed and then derived into the final variables while following the same rules as non-imputed data [39].

All analyses were performed using R (v 4.2.1).

### Sensitivity analysis

To examine the robustness of the results, sensitivity analyses were performed. We repeated the primary analyses adjusting for the alternative larger minimal sufficient adjustment set identified using the DAG. We also repeated the primary analyses using complete data without missing values (also known as listwise deletion) in place of multiple imputation. Finally, to investigate potential non-linear relationships, we compared the Akaike information criterion (AIC) of the primary models and of models where the fire-related PM_2.5_ exposure variables, which had right-skewed distributions, were log-transformed.

## Results

### Participant characteristics

A total of 103 children presenting to the 2021 clinical follow-up agreed to provide a blood sample. Children born overseas (*n* = 2) were excluded from the analyses as their background exposure at birth could not be estimated accurately, which led to the inclusion of 101 children (Fig. 2). Among them, 50 were born before the start of the fire, four were born during the fire, 29 were in utero during the whole fire period and 18 were conceived after the fire. Baseline and exposure characteristics are presented in Table 1. Within the participants, evidence of collinearity amongst the three exposures was low, with all − 0.3 < Pearson’s *r* < 0.1, with the exception from mean and peak values for fire-related PM_2.5_ at each stage (prenatal, postnatal), which were related, but included in separate models (Fig. S3).

**Table 1:** Baseline and exposure characteristics of the study participants

	Participants (***N*** = 101) n (%)
**Sex, female**	48 (47.5%)
**Maternal education, > Year 12**	71 (70.3%)
**Breastfeeding > 6 months**^**a**^	56 (55.4%)
**Main heater releasing combustion emissions into living space**^**b**^	35 (34.7%)
**Stress during pregnancy**	
No/hardly	29 (28.7%)
Sometimes/mostly	72 (71.3%)
**Premature birth**	6 (5.9%)
**Any smoker in the house**^**c**^	17 (16.8%)
**Mother with history of asthma/allergic rhinitis**^**d**^	34 (33.7%)
**Father with history of asthma/allergic rhinitis**^**e**^	35 (34.7%)
**Child has received a diagnosis of asthma**	15 (14.9%)
**Child has received a diagnosis of eczema/dermatitis**^**f**^	30 (29.7%)
	**Mean (SD)** **Median [Q1-Q3]** **Range**
**Age at blood collection (years)**	6.8 (1.0)
	7 [6–8]
	5–9
**Age at baseline (months)**	29.4 (12.6)
	28 [19–38]
	7–64
**IRSD decile**	3.9 (3.0)
	3 [1–6]
	1–10
**Average prenatal fire-related PM**_**2.5**_**(μg/m**^**3**^**)**	2.7 (7.1)
	0.0 [0.0–1.7]
	0.0–44.2
**Average postnatal fire-related PM**_**2.5**_**(μg/m**^**3**^**)**	3.7 (7.0)
	0.2 [0.0–2.5]
	0.0–30.2
**Peak prenatal fire-related PM**_**2.5**_**(μg/m**^**3**^**)**	45.4 (107.2)
	0.0 [0.0–35.7]
	0.0–593.5
**Peak postnatal fire-related PM**_**2.5**_**(μg/m**^**3**^**)**	59.5 (91.8)
	1.1 [0.0–97.0]
	0.0–447.1
**Background PM**_**2.5**_**(μg/m**^**3**^**)**	6.0 (1.0)
	6.0 [5.6–6.6]
	0.8–8.4

Note: All characteristics were assessed in the baseline questionnaire with the exception of age at blood collection, which was calculated based on their visit date at the second follow-up clinic

^a^ Missing data: n = 1. ^b^ Missing data: *n* = 3. ^c^ Missing data: *n* = 2. ^d^ Missing data: *n* = 1. ^e^ Missing data: *n* = 8. ^f^ Missing data: n = 1

### Allergen-specific sensitisation

Prevalence rates of sensitisation to each allergen in the cohort are presented in Table 2. Sensitisation to *D. pteronyssinus* had the highest prevalence (35.4%), while *Cl. herbarum* had the lowest (2.0%). There were various levels of correlation between sensitisation to allergens from different categories, the strongest being between perennial rye grass pollen and *D. pteronyssinus* (Fig. S4). We found no association between the levels of fire-related PM_2.5_ and the odds of sensitisation to any of the distinct allergen categories, for both peak and cumulative exposure (Table 3). Exposure to fire-related PM_2.5_ was not linked with sensitisation to any category either.

**Table 2:** Prevalence of sensitisation for each allergen and descriptive total IgE levels within the 101 participating children

Allergen	Number of children tested	Prevalence of sensitisation, n (%)
**Dust**		
*Dermatophagoides pteronyssinus*	99	35 (35.4%)
Dust panel	101	35 (34.7%)
**Cat**		
Cat epithelium	101	14 (13.9%)
**Grass**		
Perennial rye grass	100	34 (34.0%)
Grass panel	100	32 (32.0%)
**Fungi**		
*Alternaria tenuis*	100	10 (10.0%)
*Cladosporium herbarum*	101	2 (2.0%)
	**Number of children tested**	**Median (Q1─Q3)**
**Total IgE (kU/L)**	82	387.3 (83.9**─**816.5)

Prevalences of sensitisation were calculated with the number of children tested to that specific allergen as a denominator

**Table 3:** Association between exposure to the various sources of PM_2.5_, and sensitisation to various allergen categories and total IgE levels

**Allergen categories**	**Prenatal**		**Postnatal**	
	**Crude**	**Adjusted**	**Crude**	**Adjusted**
	**OR [95% CI]**	**OR**_**adj**_**[95% CI]**	**OR [95% CI]**	**OR**_**adj**_**[95% CI]**
(A) Average fire-related PM_2.5_				
**Dust**	0.91 [0.79,1.05]	0.93 [0.81,1.08]	1.02 [0.88,1.18]	0.95 [0.80,1.12]
**Cat**	1.00 [0.88,1.14]	1.03 [0.90,1.18]	1.12 [0.95,1.33]	1.12 [0.93,1.35]
**Grass**	0.87 [0.72,1.04]	0.91 [0.76,1.09]	1.13 [0.97,1.31]	1.06 [0.90,1.24]
**Fungi**	0.95 [0.78,1.17]	1.01 [0.84,1.22]	1.14 [0.95,1.36]	1.09 [0.89,1.32]
**Any**	0.88 [0.77,1.01]	0.90 [0.78,1.03]	1.04 [0.90,1.20]	0.96 [0.82,1.13]
	**β [95% CI]**	**β**_**adj**_**[95% CI]**	**β [95% CI]**	**β**_**adj**_**[95% CI]**
**Total IgE**	−4.6 [−25.8,16.7]	−3.9 [−25.9,18.2]	−17.5 [− 49.2,14.2]	−27.4 [− 62.3,7.5]
(B) Peak fire-related PM_2.5_				
**Dust**	0.93 [0.79,1.09]	0.96 [0.82,1.13]	1.11 [0.72,1.69]	0.87 [0.52,1.45]
**Cat**	1.05 [0.89,1.24]	1.11 [0.93,1.32]	1.47 [0.89,2.44]	1.59 [0.89,2.83]
**Grass**	0.83 [0.65,1.05]	0.89 [0.71,1.13]	1.48 [0.95,2.30]	1.19 [0.72,1.96]
**Fungi**	1.02 [0.84,1.24]	1.11 [0.90,1.36]	1.32 [0.75,2.33]	1.15 [0.58,2.26]
**Any**	0.89 [0.76,1.04]	0.91 [0.78,1.07]	1.17 [0.76,1.79]	0.90 [0.55,1.48]
	**β [95% CI]**	**β**_**adj**_**[95% CI]**	**β [95% CI]**	**β**_**adj**_**[95% CI]**
**Total IgE**	−8.1 [−39.1,22.8]	−9.8 [− 42.7,23.1]	− 51.7 [− 144.8,41.3]	− 94.9 [− 204.2,14.3]
**Allergen categories**	**Background**			
	**Crude**	**Adjusted**		
	**OR [95% CI]**	**OR**_**adj**_**[95% CI]**		
(C) Background PM_2.5_				
**Dust**	1.96 [1.16,3.29]	1.90 [1.12,3.21]		
**Cat**	1.39 [0.74,2.62]	1.38 [0.70,2.73]		
**Grass**	1.67 [1.02,2.74]	1.52 [0.92,2.51]		
**Fungi**	1.58 [0.77,3.28]	1.42 [0.70,2.84]		
**Any**	1.44 [0.94,2.21]	1.42 [0.92,2.18]		
	**β [95% CI]**	**β**_**adj**_**[95% CI]**		
**Total IgE**	39.0 [− 60.7138.6]	42.1 [−57.3141.4]		

Note: Odds ratios and 95%CI from the crude models were estimated with univariable logistic regression/linear regression models incorporating only the outcome (logistic: sensitisation to allergen category, linear: total IgE levels) and a single pollutant. Odds ratios and 95%CI from the adjusted models incorporated all three exposures and maternal education, age in months, and IRSD. All estimates were scaled by IQR increase of the relevant pollutant. *IgE* Immunoglobulin E

Early life background exposure to PM_2.5_ was positively associated with the odds of being sensitised to dust (adjusted OR = 1.90, 95%CI = 1.12,3.21), but not with cat, grass, fungi, or overall sensitisation (Table 3). The two sensitivity analyses adjusting for the larger set of possible confounders and including only complete cases without performing imputation obtained similar results for all the exposures (Tables S1-S2). Log-transformation of fire-related PM_2.5_ did not seem to improve goodness-of-fit of the models in most cases (Table S3).

### Total IgE

The median total IgE levels of children in our cohort was of 387.3 ng/ml (Table 2). Total IgE levels were moderately correlated with specific IgE to *D. pteronyssinus*, the dust panel, perennial rye grass and the grass panel (0.45 ≤ Spearman’s ρ ≤ 0.49). We did not observe evidence of a relationship between exposure to fire-related or background PM_2.5_ and overall total IgE in the blood. Both sensitivity analyses were consistent with the primary results (Tables S1-S2). Log-transformation of fire-related PM_2.5_ concentrations slightly decrease the AIC of the model, but the improvement was marginal (Table S3).

## Discussion

To the best of our knowledge, this is the first study to investigate allergic sensitisation in relation to exposure to air pollution from a landscape fire in a non-occupational setting. We did not observe a relationship between exposure in utero or during the first 2 years following birth to PM_2.5_ emitted by coal smoke and allergic sensitisation, but increased background PM_2.5_ concentrations were associated with higher prevalence of sensitisation to dust.

In terms of sensitisation to specific allergens, the only positive association was found with dust amongst the tested allergens. Studies conducted in adult populations have found conflicting results, with some observing a relationship with sensitisation to aeroallergens [40], some with indoor [41] or outdoor [42, 43] exclusively, while others found no association [44, 45]. However, our findings are consistent with the only two existing studies investigating relationships between outdoor air pollution and sensitisation to aeroallergens in Australian children. A cross-sectional study sampling 2226 children aged 7–11 years old from 12 Australian cities found that previous exposure to higher levels of NO_2_ was associated with greater odds of sensitisation to indoor allergens, particularly HDM, to which 36% of children were sensitised, a similar prevalence to the one we observed. No association was found between previous or current exposure to NO_2_ and sensitisation to fungi or grass [46]. Another cross-sectional study conducted on 382 children born in Sydney, Australia, and aged 8 years found the most consistent association to be between weighted road density and sensitisation to HDM, albeit those children having a higher rate of sensitisation (43%). A positive relationship was also found between weighted road density within 75 m radius of home and specific IgE against *Al. tenuis*, but no evidence of association was found between road density and sensitisation to cat or grass [47].

In other areas of the world, results have been less in accordance with our findings. A meta-analysis of five European birth cohorts with standardised exposure assessment did not find any evidence of associations between birth or current exposure to NO_x_, NO_2_ or PM with sensitisation to inhalant and/or food allergens at ages 4–6 or 8–10 years [48]. However, it should be noted that no distinction was made between indoor and outdoor aeroallergens in their analyses. A subsequent meta-analysis of the four largest birth cohorts also found no overall association with sensitisation to combined inhalant and food allergens in 15–16 year old children. Nonetheless, positive relationships were found between the levels of exposure at birth to PM_2.5_ and sensitisation to specific grass (*Phleum pratense* 1) and cat (*Felis domesticus* 1) allergen molecules in two of the cohorts [12]. A study conducted on a cohort of 634 American young children found that high exposure to diesel exhaust particles at age 1 was weakly associated with later sensitisation to aeroallergens at ages 2 and 3. Although, it should be noted that this cohort selected children with at least one atopic parent [49].

The discrepancy between these studies and ours could be due to variability in indoor environments between the cohorts, as gene/environment-environment interactions could play a role in the development of atopy. For example, interactions between HDM and dust exhaust particles (DEP) exposures have been observed in mouse models, where HDM specific IgE has been found to be both decreased [50] and increased [51] in HDM + DEP exposed mice in comparison mice exposed to HDM alone. Additionally, genetic polymorphisms in Glutathione S-Transferase P1 and Tumour Necrosis Factor have also been shown to modify the relationship between exposure to traffic-related air pollution and sensitisation to food and inhalant allergens in children up to 4 years old [52]. Finally, some lifestyle factors we could not adjust for, such as diet, could also affect the observed relationship between air pollution and sensitisation to allergens. In line with this, a randomised control trial found that children taking fish oil supplementation from birth had a reduced association between weighted road density and sensitisation to HDM when they reached 5 years of age in comparison with the placebo group [53].

Total IgE has been shown to be more elevated in cases of severe atopic eczema [54], allergic rhinitis [55], and asthma [56]. In our study, we did not observe association between exposure to PM_2.5_ of any source and total IgE levels, while previous studies have reported variable findings. A study conducted on 123 Iranian boys aged 6–9 years old with no history of allergy found that total IgE levels were correlated with blood levels of As, Cr, Hg, and Pb, the latter directly related to their concentration in the ambient PM_2.5_ [57]. Another study conducted in a Romanian city, where poor air quality was mainly caused by oil refineries and traffic, found a strong correlation between PM_1_ exposure and total IgE levels in 135 children aged 2–10 years old [58]. A Dutch birth cohort found no evidence of association between long-term exposure to PM_2.5_, soot or NO_2_ and raised total IgE levels in 713 children followed up to the age of 4 years old [59], while prenatal levels of black carbon and PM_2.5_ were correlated with total IgE levels in adolescence in 590 children from an American pre-birth cohort [60]. The discrepancies could be explained by divergences in pollutant types and sources, exposure windows, and methods. The median levels in our cohort were higher than other children in that age range [61], which could potentially indicate a greater atopic predisposition in our participants, compared with other children of the same age. This should be interpreted with caution as conditions such as parasitic infections may also cause elevated IgE [62], and we did not have information about this for our participants. However, it is notable that 15% of participating children were reported by their parents as having received a diagnosis of asthma by a doctor at the time of enrolment to the study (average age 29 months). This is higher that the reported prevalence of asthma in Australian children 0–4 years old of 5% [63]. While atopy is an important risk factor for the development of asthma in childhood [64], the accurate diagnosis of asthma in this young age group can be difficult [65]. Nevertheless, it is plausible that parents of children with a diagnosis of asthma would have been more likely to enrol in this study compared with parents of children without asthma, and this could have led to a sample with a higher atopic predisposition, than the wider Australian population. Periods of exposure in early life could also be of critical importance, as demonstrated in the American pre-birth cohort, as they found the association with increased exposure to black carbon during the third trimester of pregnancy to be stronger than during the first or second [60]. Larger studies investigating susceptible windows of foetal and childhood development are necessary to clarify the effect of various sources of air pollution on total IgE and other biomarkers predicting future allergic conditions.

A major strength of this study was that we were able to estimate exposure to fire-related PM_2.5_ using detailed activity data of the pregnant mother or child reported for day and night periods during the 7 weeks of the fire. Using the date of birth and gestational age, we were also able to distinguish prenatal and postnatal exposure windows and treat them separately in the analyses, as indirect and direct exposures may not be equivalent in term of toxicity. Another strength was that we were able to simultaneously assess air pollution from a severe time-limited hazard and from otherwise chronic low background levels.

However, some limitations should be acknowledged, including the limited sample size of 101 participants which restricted statistical power and did not allow us to explore stratification by established risk factors for atopy (e.g.*,* family history, breastfeeding, environmental tobacco smoke) that have been hypothesised as being possible effect modifiers for the relationship between allergy and air pollution. The large number of models tested could also have led to an increased risk of Type I error. However, we chose to not use multiple comparison as they are known to increase the type II error rate [66]. Furthermore, the loss to follow-up seemed to have disproportionately affected participants where the mothers had lower rates of post-secondary education and higher rates of maternal smoking [18, 67, 68]. No association was observed within our cohort between presence of a smoker in the house or IRSD and exposure to air pollution or sensitisation, but children with a mother with a post-secondary education had reduced odds of being sensitised to grass (Table S4). Thus, the generalisability of our results could be impacted by selection bias. Additionally, in contrast to exposure to fire-related PM_2.5_, we were only able to estimate background PM_2.5_ at the birth residence and did not have access to air pollution data between 2016 and 2021, a period that overlapped with an important bushfire season in Australia (2019–2020). However, non-differential misclassification of exposure tends to bias effect estimates towards the null [69]. Finally, IgE levels tend to increase until adolescence [34, 70] and age was directly related to exposure to smoke in our study, as unexposed children were the youngest, which might have biased our estimates away from the null. We tried to minimise this bias by incorporating age as a confounder in our analysis. Further, this correlation would not have affected the relationship with background PM_2.5_.

In conclusion, our study indicates chronic exposure to relatively low levels of air pollution in early life could have a stronger association with the development of atopy than time-limited high levels, which may be part of the mechanism relating air pollution to allergic diseases, such as allergic rhinitis and atopic dermatitis. Larger studies should examine periods of increased risk during pregnancy and infancy based on knowledge on the maturation of the immune system.

### Supplementary Information

**Additional file 1: Figure S1.** Directed acyclic graph. **Figure S2.** Total IgE distribution within our sample, (A) non-transformed (skewness=0.54), and (B) log-transformed (skewness=-1.37). **Figure S3.** Pearson’s pairwise correlation coefficients between all exposures. **Figure S4.** Spearman’s pairwise correlation coefficients between IgE concentrations (specific and total). **Table S1.** Association between exposure to the various sources of PM2.5, and sensitisation to various allergen categories and total IgE levels (adjustment for larger minimal sufficient adjustment set). **Table S2.** Association between exposure to the various sources of PM2.5, and sensitisation to various allergen categories and total IgE levels (without imputation). **Table S3.** Comparison of Akaike Information Criterion (AIC) between the primary analysis models and models with log-transformed fire-related PM2.5 concentrations. **Table S4.** Association between factors varying between the whole population of the area and the participants with exposure to PM2.5 and sensitisation.

## Data Availability

The data that support the findings of this study are available from the corresponding author upon reasonable request.

## Abbreviations

DAG: Directed acyclic graph
DEP: Diesel exhaust particles
HDM: House dust mites
IgE: Immunoglobulin E
IQR: Interquartile range
IRSD: Index of Relative Socio-economic Disadvantage
NO_2_: Nitrogen dioxide
NO_x_: Nitrogen oxides
OR: Odds ratio
PM_2.5_: Particulate matter with an aerodynamic diameter of less than 2.5 μm
RMSE: Root mean square error
SO_2_: Sulphur dioxide
