# Supplementary material for: Exposure to air pollution concentrations of various intensities in early life and allergic sensitisation later in childhood
Source: BMC Pulm Med. 2023 Dec 21;23:516. doi: 10.1186/s12890-023-02815-8 (PMC10740230; doi:10.1186/s12890-023-02815-8)
Supplement: Supplementary file 1 — Additional file 1: Figure S1. Directed acyclic graph. Figure S2. Total IgE distribution within our sample, (A) non-transformed (skewness=0.54), and (B) log-transformed (skewness=-1.37). Figure S3. Pearson’s pairwise correlation coefficients between all exposures. Figure S4. Spearman’s pairwise correlation coefficients between IgE concentrations (specific and total). Table S1. Association between exposure to the various sources of PM2.5, and sensitisation to various allergen categories and total IgE levels (adjustment for larger minimal sufficient adjustment set). Table S2. Association between exposure to the various sources of PM2.5, and sensitisation to various allergen categories and total IgE levels (without imputation). Table S3. Comparison of Akaike Information Criterion (AIC) between the primary analysis models and models with log-transformed fire-related PM2.5 concentrations. Table S4. Association between factors varying between the whole population of the area and the participants with exposure to PM2.5 and sensitisation. [file 12890_2023_2815_MOESM1_ESM.docx]

# Supplementary material


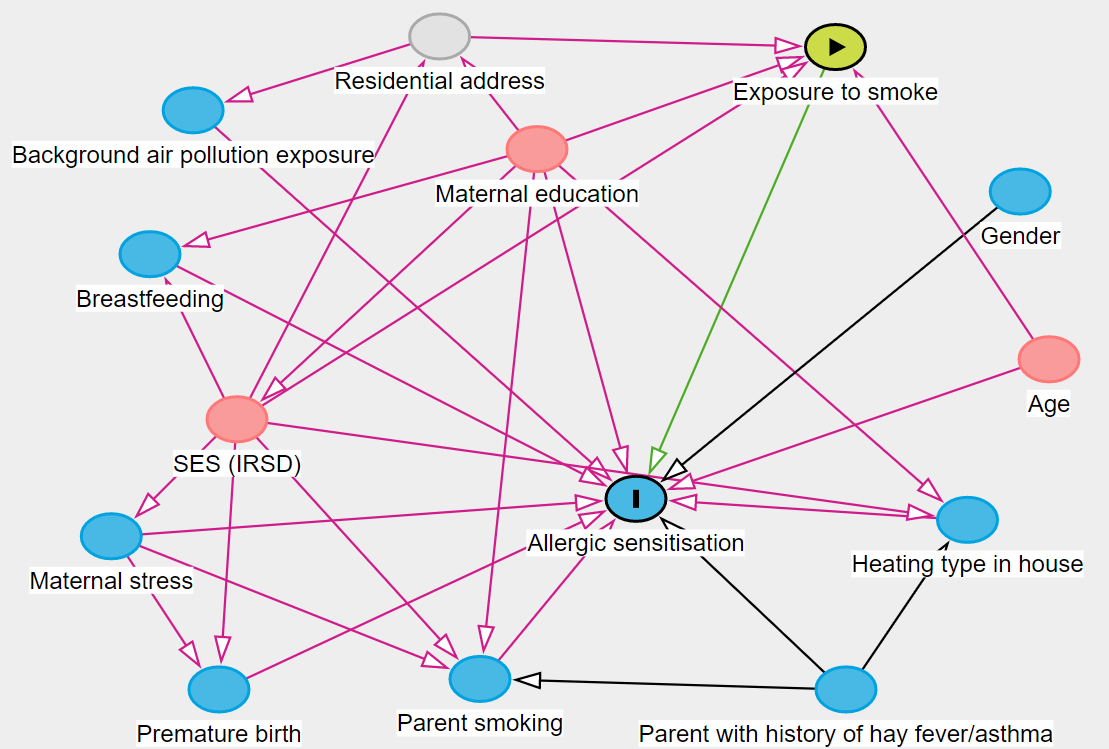


Figure S1 – Directed acyclic graph


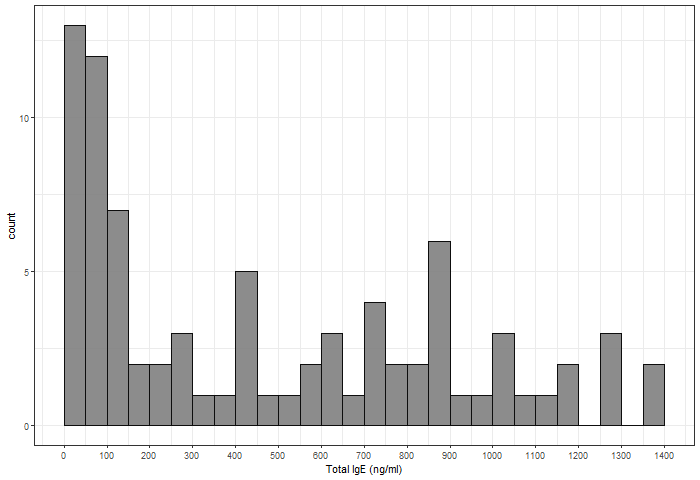


(A)


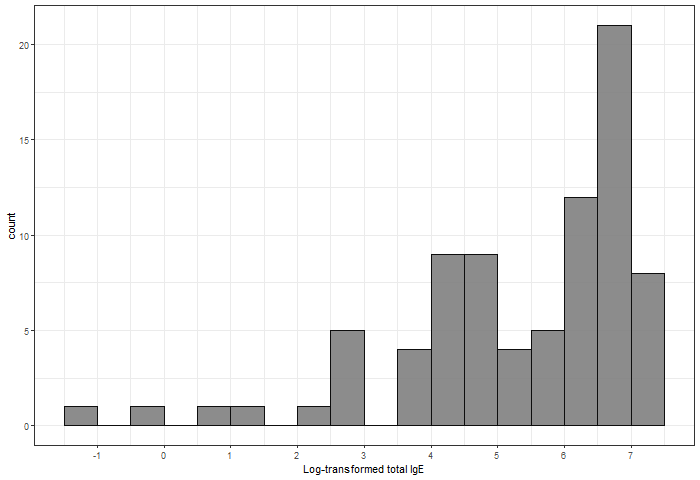


(B)

Figure S2 – Total IgE distribution within our sample, (A) non-transformed (skewness=0.54), and (B) log-transformed (skewness=-1.37).


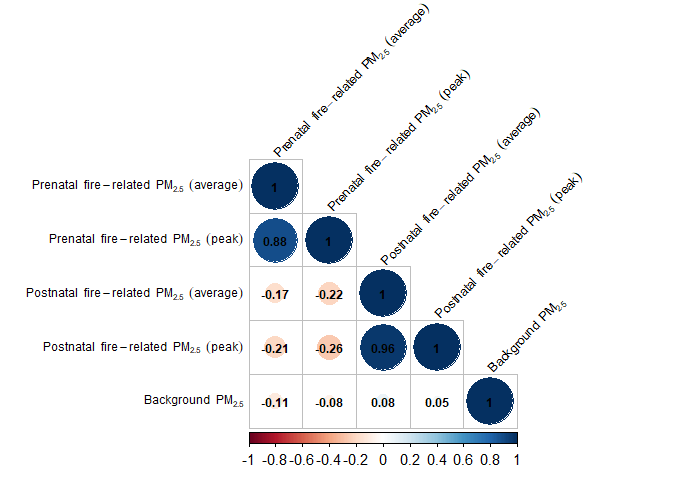


Figure S3 – Pearson’s pairwise correlation coefficients between all exposures


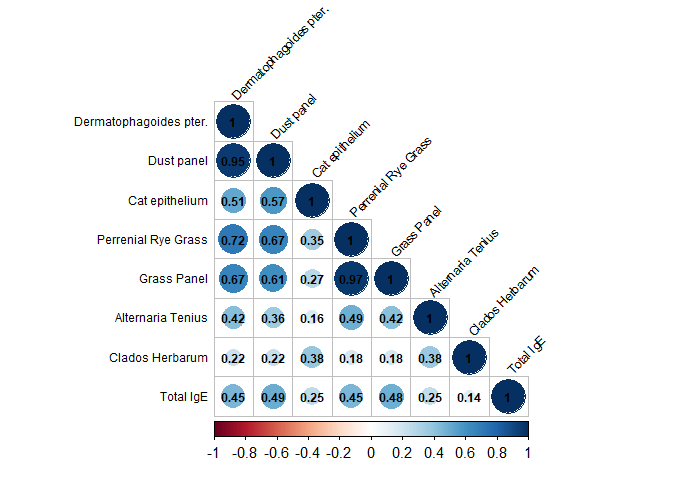


Figure S4 – Spearman’s pairwise correlation coefficients between IgE concentrations (specific and total)

Table S1 – Association between exposure to the various sources of PM_2.5_, and sensitisation to various allergen categories and total IgE levels (adjustment for larger minimal sufficient adjustment set)

1. Fire-related PM_2.5_

| **Allergen categories** | **Prenatal** | | **Postnatal** | |
| --- | --- | --- | --- | --- |
|  | **Average** | **Peak** | **Average** | **Peak** |
|  | **OR_adj_ [95% CI]** | **OR_adj_ [95% CI]** | **OR_adj_ [95% CI]** | **OR_adj_ [95% CI]** |
| **Dust** | 0.94 [0.82,1.08] | 0.96 [0.82,1.13] | 0.95 [0.80,1.13] | 0.88 [0.52,1.51] |
| **Cat** | 1.00 [0.87,1.16] | 1.07 [0.89,1.28] | 1.14 [0.93,1.39] | 1.66 [0.89,3.09] |
| **Grass** | 0.90 [0.74,1.09] | 0.87 [0.68,1.12] | 1.07 [0.91,1.27] | 1.27 [0.76,2.13] |
| **Fungi** | 1.03 [0.86,1.23] | 1.13 [0.91,1.40] | 1.12 [0.90,1.38] | 1.27 [0.60,2.66] |
| **Any** | 0.90 [0.78,1.03] | 0.91 [0.77,1.07] | 0.97 [0.82,1.15] | 0.94 [0.56,1.57] |
|  | | | | |
|  | **β_adj_ [95% CI]** | **β_adj_ [95% CI]** | **β_adj_ [95% CI]** | **β_adj_ [95% CI]** |
| **Total IgE** | -3.7 [-25.9,18.5] | -9.2 [-42.7,24.3] | -26.2 [-62.0,9.6] | -87.8 [-200.8,25.3] |

1. Background PM_2.5_

| **Allergen categories** | **PM_2.5_** |
| --- | --- |
|  | **OR_adj_ [95% CI]** |
| **Dust** | 2.03 [1.12,3.67] |
| **Cat** | 1.30 [0.63,2.70] |
| **Grass** | 1.46 [0.87,2.45] |
| **Fungi** | 1.38 [0.65,2.96] |
| **Any** | 1.33 [0.84,2.13] |
|  | **β_adj_ [95% CI]** |
| **Total IgE** | 35.6 [-67.3,138.5] |

Note: Odds ratios and 95%CI from the adjusted models were estimated with logistic regression/linear regression models incorporating the outcome (logistic: sensitisation to allergen category, linear: total IgE levels), all three exposures, breastfeeding, main heating type in the house, maternal education, pregnancy stress, presence of a smoker in the house, parent with history of asthma or allergic rhinitis, and premature birth. All estimates were scaled by IQR increase of the relevant pollutant. IgE: Immunoglobulin E.

Table S2 – Association between exposure to the various sources of PM_2.5_, and sensitisation to various allergen categories and total IgE levels (without imputation).

1. Fire-related PM_2.5_

| **Allergen categories** | **Prenatal** | | **Postnatal** | |
| --- | --- | --- | --- | --- |
|  | **Average** | **Peak** | **Average** | **Peak** |
|  | **OR_adj_ [95% CI]** | **OR_adj_ [95% CI]** | **OR_adj_ [95% CI]** | **OR_adj_ [95% CI]** |
| **Dust** | 0.93 [0.78,1.05] | 0.95 [0.79,1.11] | 0.89 [0.72,1.06] | 0.68 [0.35,1.22] |
| **Cat** | 1.03 [0.87,1.16] | 1.11 [0.91,1.31] | 1.12 [0.92,1.35] | 1.59 [0.87,2.84] |
| **Grass** | 0.91 [0.72,1.05] | 0.89 [0.66,1.08] | 1.06 [0.90,1.25] | 1.19 [0.72,1.99] |
| **Fungi** | 1.01 [0.76,1.17] | 1.11 [0.86,1.35] | 1.09 [0.87,1.31] | 1.14 [0.52,2.11] |
| **Any** | 0.90 [0.76,1.01] | 0.90 [0.75,1.05] | 0.92 [0.77,1.10] | 0.76 [0.42,1.34] |
|  | | | | |
|  | **β_adj_ [95% CI]** | **β_adj_ [95% CI]** | **β_adj_ [95% CI]** | **β_adj_ [95% CI]** |
| **Total IgE** | -5.9 [-29.4,17.7] | -17.1 [-52.9,18.6] | -30.6 [-68.4,7.2] | -118.5 [-239.7,2.6] |

1. Background PM_2.5_

| **Allergen categories** | **PM_2.5_** |
| --- | --- |
|  | **OR_adj_ [95% CI]** |
| **Dust** | 1.87 [1.16,3.31] |
| **Cat** | 1.38 [0.75,2.86] |
| **Grass** | 1.50 [0.95,2.58] |
| **Fungi** | 1.41 [0.77,3.03] |
| **Any** | 1.41 [0.94,2.25] |
|  | **β_adj_ [95% CI]** |
| **Total IgE** | 37.3 [-65.2,139.7] |

Note: Odds ratios and 95%CI from the adjusted models were estimated with logistic regression/linear regression models incorporating the outcome (logistic: sensitisation to allergen category, linear: total IgE levels), all three exposures, maternal education, age in months, and IRSD. All estimates were scaled by IQR increase of the relevant pollutant. IgE: Immunoglobulin E.

Table S3 – Comparison of Akaike Information Criterion (AIC) between the primary analysis models and models with log-transformed fire-related PM_2.5_ concentrations.

| **Outcome category** | **AIC main model (A)** | **AIC after log-transformation (B)** | **Differences in AIC (B-A)** |
| --- | --- | --- | --- |
| **Dust** | 135.20 | 133.93 | -1.27 |
| **Cat** | 92.37 | 93.25 | 0.88 |
| **Grass** | 131.61 | 132.64 | 1.03 |
| **Fungi** | 78.46 | 78.86 | 0.40 |
| **Any** | 143.57 | 142.92 | -0.65 |
| **Total IgE** | 1514.85 | 1512.81 | -2.05 |

Table S4 – Association between factors varying between the whole population of the area and the participants with exposure to PM2.5 and sensitisation.

1. Presence of a smoker in the house

| **Exposures/sensitisation** | **β [95% CI]** |
| --- | --- |
| **Prenatal average fire-related PM_2.5_** | -1.34 [-3.58,0.91] |
| **Postnatal average fire-related PM_2.5_** | 0.22 [-1.24,1.69] |
| **Prenatal peak fire-related PM_2.5_** | -0.90 [-2.48, 0.68] |
| **Postnatal peak fire-related PM_2.5_** | 0.04 [-0.46,0.54] |
| **Background PM2.5** | 0.25 [-0.32,0.83] |
| **Total IgE** | 138.18 [-93.72,370.09] |
|  | **OR [95% CI]** |
| **Dust** | 1.63 [0.56,4.74] |
| **Cat** | Did not converge |
| **Grass** | 1.42 [0.48,4.22] |
| **Fungi** | 1.20 [0.23,6.37] |
| **Any** | 1.51 [0.52,4.42] |

Note: Odds ratios and 95%CI were estimated with logistic regression/linear regression models with presence of a smoker in the house (yes/no) as the predictor and the various exposures and measures of sensitisation as outcomes. IgE: Immunoglobulin E.

1. IRSD

| **Exposures/sensitisation** | **β [95% CI]** |
| --- | --- |
| **Prenatal average fire-related PM_2.5_** | 0.20 [-0.08,0.48] |
| **Postnatal average fire-related PM_2.5_** | -0.12 [-0.31,0.06] |
| **Prenatal peak fire-related PM_2.5_** | 0.16 [-0.04,0.36] |
| **Postnatal peak fire-related PM_2.5_** | -0.05 [-0.12,0.01] |
| **Background PM2.5** | -0.03 [-0.10,0.04] |
| **Total IgE** | 0.33 [-30.04,30.70] |
|  | **OR [95% CI]** |
| **Dust** | 0.99 [0.86,1.13] |
| **Cat** | 0.98 [0.81,1.19] |
| **Grass** | 0.92 [0.80,1.07] |
| **Fungi** | 0.97 [0.78,1.21] |
| **Any** | 1.01 [0.88,1.15] |

Note: Odds ratios and 95%CI were estimated with logistic regression/linear regression models with IRSD decile as the predictor and the various exposures and measures of sensitisation as outcomes. IgE: Immunoglobulin E.

1. Maternal education

| **Exposures/sensitisation** | **β [95% CI]** |
| --- | --- |
| **Prenatal average fire-related PM_2.5_** | 1.07 [-0.78,2.91] |
| **Postnatal average fire-related PM_2.5_** | -0.80 [-2.00,0.39] |
| **Prenatal peak fire-related PM_2.5_** | 0.85 [-0.44,2.14] |
| **Postnatal peak fire-related PM_2.5_** | -0.30 [-0.71,0.11] |
| **Background PM2.5** | -0.31 [-0.77,0.14] |
| **Total IgE** | -20.01 [-215.29,175.27] |
|  | **OR [95% CI]** |
| **Dust** | 0.87 [0.36,2.10] |
| **Cat** | 0.73 [0.22,2.42] |
| **Grass** | 0.39 [0.16,0.96] |
| **Fungi** | 0.71 [0.19,2.68] |
| **Any** | 0.70 [0.30,1.68] |

Note: Odds ratios and 95%CI were estimated with logistic regression/linear regression models with maternal education (≤ year 12 vs. > year 12) as the predictor and the various exposures and measures of sensitisation as outcomes. IgE: Immunoglobulin E.
